# Youth Sports Participation Is More Important among Females than Males for Predicting Physical Activity in Early Adulthood: Iowa Bone Development Study

**DOI:** 10.3390/ijerph18031328

**Published:** 2021-02-02

**Authors:** Soyang Kwon, Elena M. Letuchy, Steven M. Levy, Kathleen F. Janz

**Affiliations:** 1 Ann & Robert H. Lurie Children’s Hospital of Chicago, Chicago, IL 60611, USA; 2 Department of Epidemiology, University of Iowa, Iowa City, IA 52242, USA; elena-letuchy@uiowa.edu (E.M.L.); steven-levy@uiowa.edu (S.M.L.); kathleen-janz@uiowa.edu (K.F.J.); 3 Department of Preventive & Community Dentistry, University of Iowa, Iowa City, IA 52242, USA; 4 Department of Health and Human Physiology, University of Iowa, Iowa City, IA 52242, USA

**Keywords:** accelerometers, physical activity, DXA, adiposity, children and adolescents, athletics

## Abstract

A gender difference in youth sports and physical activity participation is well documented. However, research is limited to understand potential gender difference in the long-term effects of youth sports participation. The study aim was to compare the likelihood of meeting the aerobic Physical Activity Guidelines (PAG) in early adulthood by youth sports participation patterns among females and males. The study sample included 582 Iowa Bone Development Study participants (Iowa, USA). Participation in organized sports was assessed using a physical activity questionnaire 19 times on average between age 6 and 17 years. Accelerometer and dual-energy X-ray absorptiometry assessments were conducted at an average age of 23 years. Group-based trajectory analysis was conducted to identify youth sports participation trajectory patterns. Multivariable logistic regression analysis was conducted to examine the association between youth sports participation patterns and meeting the PAG in adulthood. We identified three youth sports participation patterns: “continuous participation,” “drop-out,” and “no participation.” Females in the continuous participation group were more likely to meet the aerobic PAG at age 23 years, compared to females in the no-participation group (OR = 2.63; 95% CI = 1.05, 6.55) or the drop-out group (OR = 2.55; 95% CI = 1.38, 4.69). However, among males, youth sports participation patterns were not significantly associated with meeting the aerobic PAG at age 23 years. In conclusion, this study suggests that youth sports participation could be more important among females than males for predicting physical activity in early adulthood.

## 1. Introduction

Physical activity provides numerous health benefits, including prevention of obesity and type 2 diabetes [1]. However, a substantial proportion of the U.S. population is physically inactive [2]. Moreover, females are less active than males even prior to kindergarten [3], and remain so throughout childhood and adulthood [2]. To ensure that females receive the same physical activity-related health benefits as males, numerous physical activity promotion strategies targeting girls have been proposed, including participation in sports [4]. Although most youth in the U.S. initiate sports participation, girls have a lower rate of sports participation than boys [4,5]. Furthermore, the majority of youth stop participating in sports over time, with a larger drop-out among girls [6,7,8,9]. Gender disparities in youth sports participation are partly associated with societal factors [10], such as the unfair share of athletic opportunities for girls as compared to boys [11] and parental attitudes toward female participation in youth sports [12].

Youth sports participation not only contributes to concurrently increasing physical activity [13,14,15], but may also contribute to sustaining healthy physical activity behaviors later in adulthood [16,17,18]. The follow-up study of the Princeton School District Study [16] showed that continuous sports participation in the middle and high school years was associated with higher physical activity, lower body mass index (BMI) and higher high-density lipoprotein cholesterol (HDL-C) in early adulthood among both males and females. In sex-specific analyses, the Cardiovascular Risk in Young Finns Study [19] revealed that the association between continuous youth sports participation and adulthood metabolic syndrome was stronger among females than males. Therefore, it is also plausible that the association between youth sports participation and adulthood physical activity is stronger among females than males, possibly because competence and confidence in movement skills developed through youth sports participation, also called physical literacy [4], can help reduce barriers to engaging in physical activity among females later in adulthood. However, the gender differences in the association between youth sports participation and adulthood physical activity have rarely been examined. Evidence for the long-term benefits of youth sports participation will strengthen evidence-based policy discussions and planning efforts that aim to promote youth sports participation and reduce the gender disparities in sports and physical activity participation.

In an earlier study of youth sports participation trajectories, we found three distinct trajectory patterns: continuous participation, drop-out, and no- to low-participation (no participation, hereafter) [7]. Expanding on those findings, the primary aim of this study was to compare the likelihood of meeting the aerobic Physical Activity Guidelines (PAG) [1] in early adulthood by the three youth sports participation patterns among females and males. We hypothesized that the association between continuous sports participation in youth and meeting the PAG in early adulthood is stronger among females than among males. The secondary aim was to describe physical activity levels in childhood by the three youth sports participation patterns among females and males. As a secondary outcome, we also explored adiposity by the three youth sports participation patterns.

## 2. Methods

### 2.1. Study Sample

The study sample was participants in the Iowa Bone Development Study (IBDS), an ongoing longitudinal study of bone health during childhood, adolescence, and early adulthood. IBDS participants were recruited from a subset of the Iowa Fluoride Study birth cohort that included 1882 full-term newborns recruited from 8 hospital postpartum wards in Iowa, USA between 1992 and 1995. The baseline examinations for the IBDS were conducted at child approximate age 5 years. Self-identified race was mostly white (95%). Additional information about the IBDS design and participants is available elsewhere [20,21]. For the current report, participants who reported sports participation at least four times over at least three years [19] between age 6 and 17 years were eligible (*n* = 582). Written informed consent and assent were obtained. The IBDS protocol was approved by The University of Iowa Institutional Review Board (199112665).

### 2.2. Measurements

#### 2.2.1. Sports Participation

The main exposure variable was youth sports participation. As the National Youth Sports Strategy (NYSS), the first federal roadmap for youth sports participation promotion, focuses on youth ages 6 to 17 years [4], we examined longitudinal sports participation from age 6 to 17 years. Because the definition of sports by the NYSS [4] encompasses not only competitive sports, but also recreational sports that are played as a team or an individual, we examined participation in organized sports (defined as coached sports or lessons), regardless of whether it was competitive (e.g., interscholastic sports) or recreational (e.g., swimming lessons).

Sports participation was assessed using a physical activity questionnaire (PAQ). The PAQ was sent via mail or administered during a research clinic visit every six months. Over time, two versions of the PAQ were used. The first version (proxy-PAQ), designed for a parent to report on a child’s activities, was used for participants younger than 11 years. The proxy-PAQ asked the following question: “Did your child participate in any of the following organized sports during the past 6 months?” Responses included baseball, basketball, soccer, gymnastics, dance, swimming, and other. Responses of “other” were reviewed to capture any other sports that were not listed. Children who were reported as having participated in at least one organized sport in the past 6 months were categorized as participating in sports. The second version (self-PAQ), adapted from the PAQs for older children and adolescents [22,23], was used for participants aged 11 years or older. The self-PAQ listed 20+ sports and asked about the frequency of participation in the previous 7 days and whether the sports activity was organized (yes/no). Responses of “other” sports were reviewed to capture any other sports that were not listed. If a respondent reported participating in at least one organized sport at least once a week, the respondent was categorized as participating in sports. We considered the following sports to be plausible in the U.S. Midwest setting: baseball, softball, basketball, cheerleading, dance, football, golf, gymnastics, tumbling, ice hockey, figure skating, martial arts, track and field running, soccer, swimming, tennis, badminton, racquetball, volleyball, lacrosse, and wrestling [7].

#### 2.2.2. Accelerometry

To directly measure physical activity, accelerometry assessments were conducted using ActiGraph accelerometers (ActiGraph LLC.; Pensacola, FL, USA) at approximate ages 5, 8, 11, 13, 15, 17, 19, 21, 23, and 25 years (waves 1 to 10, respectively) from 1998 to 2018. ActiGraph model 7164 was used for waves 1–4, GT1M for wave 5, and GT3X+ for waves 6 to 10. When participants visited the research clinic, they were given instructions for accelerometer wear. Accelerometers were mailed to participants during the autumn (September–November). The detailed procedures for accelerometer data collection were described in previous publications [7,21]. Briefly, participants were asked to wear an accelerometer during waking hours for 4 consecutive days (including 1 weekend day) during waves 1 and 2, and for 5 consecutive days (including both weekend days) for all other waves. In the accelerometry data reduction process, data during monitor wear times were selected based on the wear/non-wear time definition by the Choi et al. [24]. Next, we identified and included participants who had at least 3 valid days of accelerometer data in each wave, where a valid day was defined as having at least 10 h of valid wear time for analysis [25]. In accordance with the intensity-based cut-points for youth [26,27], moderate intensity was defined as 2296–4011 counts per minute and vigorous intensity was defined as ≥4012 counts per minute. Although adult cut-points [2] are slightly different, we applied the youth cut-points to accelerometer data collected in adulthood, so that an identical intensity definition could be used within the study sample across all waves.

Moderate- and vigorous-intensity physical activity (MVPA) from age 5 to 17 years was examined using accelerometer data collected in waves 1 to 6. Daily times spent in MVPA (minutes/day) per wave were calculated by summing daily minutes spent in moderate-intensity physical activity (MPA; minutes/day) and daily minutes spent in vigorous-intensity physical activity (VPA; minutes/day). Whether meeting the aerobic PAG [1] in adulthood was examined using accelerometer data collected between age 18 and 25 years. For participants who completed multiple accelerometer assessments between age 18 and 25 years, the latest assessment was used as the adulthood physical activity measure. Daily MPA-equivalent minutes [28] were calculated by adding daily MPA minutes and twice daily VPA minutes (MPA minutes +2 × VPA minutes). Weekly MPA-equivalent minutes (minutes/week) were estimated as 7 times the average daily MPA- equivalent minutes (7 × daily MPA equivalent minutes) obtained from 3 to 5 valid days [28]. Those with weekly MPA-equivalent minutes ≥150 min were considered as meeting the aerobic PAG in adulthood.

#### 2.2.3. Dual-Energy X-ray Absorptiometry (DXA)

Participants underwent whole-body DXA scans during research clinic visits at waves 1 to 7, and at wave 9. Pregnant females were excluded from the DXA examination. Hologic DXA models were upgraded over time: The Hologic QDR2000 model (Hologic Inc., Bedford, MA, USA) was used at waves 1 and 2, Hologic QDR4500 Delphi A model at waves 3 to 6, and Discovery A model at waves 7 and 9. The differences in fat mass estimation between different models were adjusted, as described elsewhere [29]. The scan images were analyzed using APEX software version 4.0 with the NHANES body composition analysis (BCA) option. The BCA option adds 5% of lean mass to the fat mass, to correct for the underestimation of fat mass by this particular densitometer [30]. From this analysis, total fat mass was derived. Fat mass index (FMI; kg/m^2^) was calculated as total fat mass (kg) divided by height squared (m^2^).

Adiposity from age 5 to 17 years was examined using DXA-derived FMI collected in waves 1 to 6. Among the multiple DXA assessments collected between age 18 and 23 years, the latest assessment was used as the adulthood adiposity measure. Based on sex- and age-specific FMI percentile references [31], adulthood FMI ≥ 75th percentile was considered as excess body fat [32].

#### 2.2.4. Other Measurements

Household income and mother’s education levels were reported in a mailed-in demographic survey in 2007 (approximate child age of 13 years). Maternal education level was dichotomized into <college graduation and ≥college graduation. For those without maternal education data from the 2007 survey, maternal education levels examined at earlier years were used instead. Family income level was dichotomized (lower vs. higher) based on the median value. Baseline BMI was calculated (as weight in kg divided by height squared in m^2^) using anthropometry data assessed at wave 1. Those with BMI ≥ 95th percentile were considered as obese at baseline, based on the 2000 U.S. CDC growth charts [33]. At wave 2, fathers and mothers reported their weekly frequencies of participation in specific high school sports. A parent high school sports score was calculated by multiplying the intensity of the sports (metabolic equivalents; METs) [34] by the frequency of sports participation.

### 2.3. Statistical Analysis

Based on our prior investigation [7] and other research [9], we assumed that there were three distinct trajectory patterns (groups) of youth sports participation. As such, we conducted group-based trajectory analysis to fit three regression models (binary logit distribution) for sports participation from age 6 to 17 years, using STATA TRAJ (StataCorp LP; College Station, TX, USA). To take advantage of the availability of 10+ repeated assessments, biquadratic models were initially fit for all three groups. We then reduced the level of polynomial functions for each group (biquadratic, cubic, quadratic, linear, or constant) until a parameter estimate in the highest function had a significance of *p* < 0.01. Each participant was assigned to one of the three trajectory groups based on the maximum posterior probability assignment rule [7]. The model adequacy was evaluated using multiple diagnostic measures, such as posterior probability of group classification and odds of correct classification [7].

The following analyses were conducted using SAS 9.4 (SAS Institute Inc.; Cary, NC, USA). Descriptive analysis was conducted to describe participant characteristics of the three youth sports participation groups. Chi-square tests and Analyses of Variance (ANOVA) were conducted to compare the three groups. The means of MVPA and FMI measured at waves 1 to 6 were calculated for each of the three groups, separately for females and males. Sex-specific mixed models were used to compare repeatedly-measured MVPA and FMI for the three groups, accounting for within-subject random effects.

Chi-square tests were conducted to compare MPA-equivalent minutes and FMI in adulthood among the three groups. Analyses of variance (ANOVA) were conducted to compare the proportions of those who met the aerobic PAG and had excess body fat in adulthood, respectively, among the three groups. Sex-specific multivariable logistic regression models were fit to compare the probability of meeting the aerobic PAG in adulthood among the three groups, adjusted for age (in years), household income (higher vs. lower), and baseline MVPA (minutes/day). The significance level was set at 0.05 (two-sided).

## 3. Results

**Figure 1:**
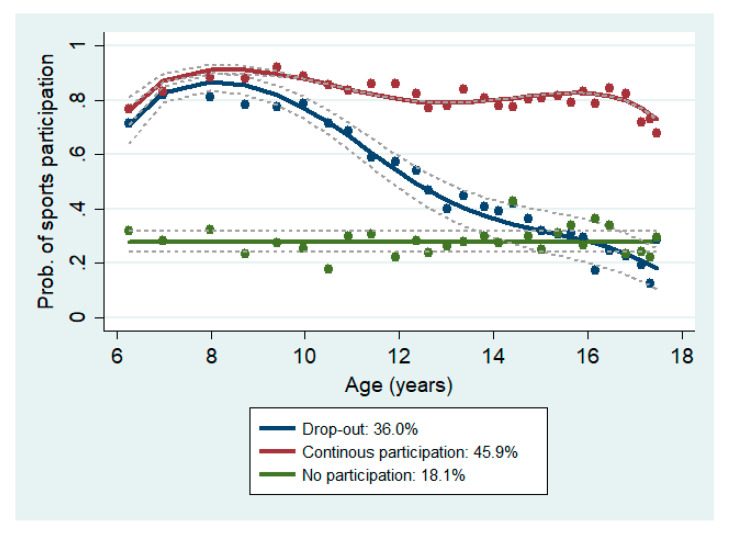
Sports participation trajectory patterns between age 6 and 17 years (*n* = 582). Note: Dots indicate actual rates of sports participation, solid lines indicate probabilities of sports participation estimated from trajectory regression models, and dotted lines indicate 95% confidence intervals of the estimated probabilities. The legend presents pattern labels and the proportion of the sample that belongs to the pattern.

A total of 582 IBDS participants (296 females) responded to the sports participation question at least four times over at least three years between age 6 and 17 years. The median number of available sports participation response points between age 6 and 17 years per individual was 19 (range of 4 to 30). The median follow-up period was 11 years (interquartile range of 8 to 12 years). As shown in Figure 1, 45.9% of the study sample followed the “continuous participation” pattern, 36.0% followed the “drop-out” pattern, and 18.1% followed the “no participation” pattern. In the drop-out pattern, the highest amount of drop-out occurred between age 9 and 13 years.

The distribution of sports participation patterns did not differ by gender (Table 1). However, higher mother’s education and higher household income were associated with consistent sports participation (*p* < 0.01). Overall, 13.4% of the participants (11.8% in females and 15.2% in males) were categorized as obese at baseline. Obesity rate at baseline was the lowest in the continuous sports participation group, followed by the drop-out group and the no-participation group among both females and males (*p* < 0.05). The parents of those in the continuous sports participation group reported the highest levels of sports participation in their high school years (*p* < 0.01).

**Figure 2:**
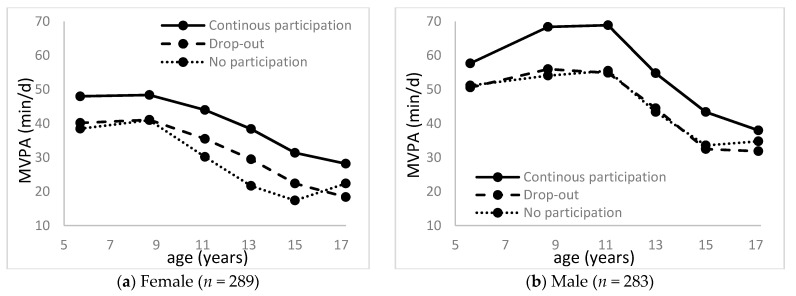
Moderate- and vigorous-intensity physical activity (MVPA) during childhood by youth sports participation patterns: (1) female and (2) male.

Of the 582 participants, 572 (289 females) completed at least one accelerometer assessment between age 5 and 17 years. Mean MVPA minutes from age 5 to 17 years are illustrated in Figure 2. Mixed regression models showed that the mean MVPA was higher in the continuous sports participation group than in the no-participation or drop-out group among both females and males (*p* < 0.05). There were no significant differences in MVPA between the no-participation and drop-out groups.

**Figure 3:**
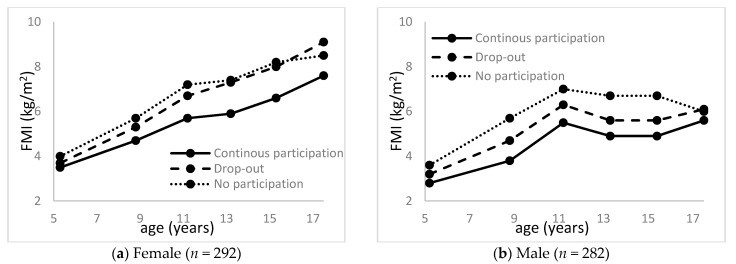
Fat mass index (FMI) during childhood by youth sports participation: (**a**) female and (**b**) male.

Of the 582 participants, 574 (292 females) completed at least one DXA assessment between age 5 and 17 years. Mean FMIs from age 5 to 17 years are illustrated in Figure 3. Mixed regression models showed that the mean FMI was lower in the continuous sports participation group than in the no-participation or drop-out group among both females and males (*p* < 0.05). Compared to the no-participation group, the drop-out group had lower FMI among males (*p* < 0.05), but not among females.

Of the 582 participants, 407 (223 females) completed at least one accelerometer assessment in adulthood. The median age at the last accelerometer assessment was 23.7 years (interquartile range of 22.9 to 25.1) and ages were similar across the three sports participation groups. Among females, but not among males, average MPA-equivalent minutes were more than twice as high in the continuous sports participation group, compared to the no-participation group (*p* < 0.01; Table 2). Among females, but not among males, the rate of meeting the aerobic PAG in adulthood was significantly higher in the continuous participation group than the other two groups. Of the 582 participants, 408 (220 females) completed at least one DXA assessment in adulthood. The median age at the last DXA assessment was 23.1 years (interquartile range of 22.8 to 23.6) and ages were similar across the three sports participation groups. Among females, but not among males, the rate of excess body fat in adulthood was significantly different: the lowest in the continuous participation group, followed by no-participation group and the drop-out group. However, when the rate of excess body fat was examined only among females who did not have obesity at baseline, the differences among the groups became statistically insignificant (*p* = 0.08).

Multivariable logistic regression models showed that, after adjusting for age, baseline MVPA, and household income, females in the continuous participation group were more likely to meet the aerobic PAG in adulthood, compared to females in the no-participation group (odds ratio = 2.63; 95% confidence interval = 1.05, 6.55; Table 3) or in the drop-out group (odds ratio = 2.55; 95% confidence interval = 1.38, 4.69). However, among males, youth sports participation patterns were not associated with meeting the aerobic PAG in adulthood.

## 4. Discussion

This study examined the associations between youth sports participation trajectory patterns and subsequent adulthood PA, focusing on a potential gender difference in the strength of the association. The study sample, which included mostly white participants from a relatively higher socioeconomic background showed no gender difference in youth sports participation. However, females were less active than males in childhood and adulthood. Both females and males who followed a continuous youth sports participation pattern presented higher MVPA and lower FMI during childhood, compared to those who followed a no-participation or drop-out pattern. However, females with continuous youth sports participation were twice as likely to meet the aerobic PAG later in adulthood, while among males, youth sports participation patterns were not associated with meeting the aerobic PAG in adulthood.

Our finding of no gender difference in sports participation patterns is contradictory to prior national studies [4,5,9] that reported a gender disparity in youth sports participation. Sociodemographic characteristics of the study sample (mostly white race and high socioeconomic status) could partly explain the finding, though not entirely, given that the national data [9] showed that the gender difference exists even within a high socioeconomic group and among white participants. Considering some of the known reasons (i.e., cost, access, and safety) for gender disparities in sports participation [9], the specific U.S. midwestern rural areas from which the study sample was drawn could have more affordable cost of sports, better access to sports programs, and greater safety, compared to other U.S. areas. Our finding demonstrates that the gender disparity in youth sports participation is not due to a biological difference, and as such it can be mitigated, presumably by providing a more supportive environment. As we found that the age of the greatest rate of drop-out was approximately 9 to 13 years, consistent with prior studies [6,9], specific interventions and policy efforts should be considered to support continuous sports participation of youth in this age.

This study found that young children with obesity were less likely to initiate sports participation. And, even if they initiated participation, they were more likely to drop out than those who were not obese. Although this finding could be confounded by the effect of socioeconomic status, because both obesity and lower sports participation have been associated with lower socioeconomic status, as shown in the current study as well as prior studies [35,36,37], it is still critical to encourage and provide sports participation opportunities to young children with or at risk of obesity.

Consistent with prior studies [13,14,15], this study supports the positive association between continuous sport participation and concurrent MVPA level. The novel finding of this study is that continuous youth sport participation is associated with physical activity in early adulthood in females, but not in males. The effect size appears to be clinically significant for females, given that the proportion of females who met the aerobic PAG in adulthood was more than twice as high in the continuous participation group than in the no-participation group (62% vs. 30%). This finding suggests that youth sports participation has long-term effects, particularly among females. For example, pre-diabetes is increasingly common in young adults, with 24.0% prevalence overall among U.S. young adults aged 19 to 34 years, and substantially higher prevalence (36.9%) among those with obesity [38]. Youth sport participation among females could help to maintain an active lifestyle for the prevention of obesity and diabetes in early adulthood. This finding also supports the recent national effort to promote youth sports participation, with a specific focus on girls, to reduce the gender disparities in sports participation [4].

Lastly, it is worth noting that a substantial difference in the aerobic PAG for youth aged 6–17 years (one hour daily [7 h weekly]) vs. adults aged 18–74 years (2.5 h weekly) [39] would have significant implications for young adults. This study found that when the adult PAG was applied, 56% of young adult participants met the aerobic PAG, while when the youth PAG was applied, only 4% met the aerobic PAG. This abrupt change of the aerobic PAG in transition from youth to adult should be addressed to provide clear goals for physical activity promotion planning among young adults.

A primary strength of this study is that it prospectively examined youth sports participation patterns over 11 years, representing the most frequently assessed youth sports participation data ever reported. Another strength is that it included the use of state-of-the-art measurements, such as sensor-based physical activity measures and DXA-derived adiposity measures. Several limitations should also be acknowledged. First, to determine whether weekly MPA equivalent minutes were ≥150 min in adulthood, we imputed physical activity data measured for 3–5 days to represent 7-day data. This imputation may have caused non-differential bias. Second, despite our efforts to adjust for DXA data that were derived from different densitometer models, we caution that the absolute fat mass values derived from the Hologic QDR2000 model used at waves 1 and 2 may not be comparable with those from later models. Third, unmeasured confounders that affect early adulthood physical activity (e.g., participant education level, peer influence, and/or social environment) could have biased our findings. Finally, the findings from this racially/ethnically and socioeconomically homogenous study population are not generalizable to other populations.

In conclusion, this study suggests that youth sports participation is more important among females than males for predicting physical activity in early adulthood. This study supports promotion efforts for female youth sports participation to reduce a gender disparity in youth sports participation and increase female engagement in physical activity in childhood and adulthood.

## Figures and Tables

**Table 1:** Participant characteristics by youth sports participation patterns (*n* = 582).

	Continuous Participation	Drop-Out	No Participation	*p*-Value
	*n* (%)	*n* (%)	*n* (%)	
Total	271 (46.6)	211 (36.2)	100 (17.2)	
Gender				0.97
Female	139 (47.0)	107 (36.1)	50 (16.9)	
Male	132 (46.1)	104 (36.4)	50 (17.5)	
Mother’s education				<0.01
<College graduation	82 (39.6)	67 (32.4)	58 (28.0)	
≥College graduation	189 (50.4)	144 (38.4)	42 (11.2)	
Family income				<0.01
Lower	129 (38.9)	124 (37.3)	79 (23.8)	
Higher	142 (56.8)	87 (34.8)	21 (8.4)	
Baseline obesity status ^a^				
Obesity in females	8 (6.3)	15 (14.6)	10 (20.0)	0.02
Obesity in males	11 (9.4)	18 (18.8)	10 (22.7)	0.05
	Mean ± SD	Mean ± SD	Mean ± SD	
Mother’s high-school sports participation score ^b^	7.5 ± 7.3	5.9 ± 7.0	3.5 ± 5.8	<0.01
Father’s high-school sports participation score ^c^	11.2 ± 7.9	8.0 ± 7.2	6.2 ± 7.8	<0.01

SD, standard deviation; ^a^ missing *n* = 45; ^b^ missing *n* = 32; ^c^ missing *n* = 160.

**Table 2:** Physical activity and adiposity in adulthood by youth sports participation patterns.

	All	Continuous Participation	Drop-Out	No Participation	*p*-Value
	Mean ± SD	Mean ± SD	Mean ± SD	Mean ± SD	
MPA equivalent, min/wk					
Females (*n* = 223)	220 ± 232	291 ± 261	167 ± 194	140 ± 154	<0.01
Males (*n* = 184)	297 ± 248	347 ± 259	339 ± 285	264 ± 262	0.63
MVPA, min/d					
Females (*n* = 223)	20 ± 17	25 ± 17	17 ± 17	14 ± 12	<0.01
Males (*n* = 184)	28 ± 21	29 ± 20	28 ± 19	26 ± 25	0.70
FMI, kg/m^2^					
Females (*n* = 223)	10.3 ± 4.8	9.4 ± 4.0	11.3 ± 5.3	10.5 ± 5.3	0.02
Males (*n* = 184)	7.3 ± 3.6	6.9 ± 3.1	7.8 ± 3.7	7.3 ± 4.3	0.28
	***n* (%)**	***n* (%)**	***n* (%)**	***n* (%)**	
Meeting the aerobic PAG					
Females (*n* = 223)	107 (48.2)	63 (61.8)	33 (36.3)	9 (30.0)	<0.01
Males (*n* = 184)	127 (69.0)	63 (71.6)	40 (62.5)	20 (62.5)	0.42
Excess body fat ^a^					
Females (*n* = 220)	70 (31.8)	25 (23.8)	34 (40.5)	11 (35.5)	0.04
Males (*n* = 188)	63 (33.5)	26 (27.1)	27 (40.9)	10 (38.5)	0.16
Females without baseline obesity (*n* = 187)	51 (27.3)	19 (20.2)	24 (35.8)	8 (30.8)	0.08
Males without baseline obesity (*n* = 148)	44 (29.7)	18 (23.4)	19 (38.0)	7 (33.3)	0.20

^a^ Defined as FMI ≥ 75th percentile; FMI, fat mass index; MPA, moderate-intensity physical activity; MVPA, moderate- and vigorous-intensity physical activity; PAG, Physical Activity Guidelines; SD, standard deviation.

**Table 3:** Multivariable logistic regression models for meeting the aerobic Physical Activity Guidelines in adulthood.

	Female (*n* = 223)	Male (*n* = 184)
	OR (95% CI)	OR (95% CI)
Age in years	0.87 (0.73, 1.05)	0.96 (0.80, 1.15)
MVPA at baseline, min/d	1.01 (1.00, 1.03)	1.01 (1.00, 1.02)
Household income		
Lower	Reference	Reference
Higher	2.55 (1.38, 4.69)	1.96 (1.00, 3.83)
Youth sports participation pattern		
No participation	Reference	Reference
Drop-out	1.03 (0.41, 2.59)	0.88 (0.35, 2.20)
Continuous participation	2.63 (1.05, 6.55)	1.28 (0.52, 3.14)

MVPA, moderate- and vigorous-intensity physical activity; OR, odds ratio; CI, confidence interval.
